# Supplementary material for: Unveiling Pharmacological Mechanisms of Bombyx mori (Abresham), a Traditional Arabic Unani Medicine for Ischemic Heart Disease: An Integrative Molecular Simulation Study
Source: Pharmaceutics. 2025 Feb 24;17(3):295. doi: 10.3390/pharmaceutics17030295 (PMC11944354; doi:10.3390/pharmaceutics17030295)
Supplement: Supplementary file 1 [file pharmaceutics-17-00295-s001.zip › Supplementary Data S1 - Computing Power.pdf]

Supplementary Data S1

Computing Power

All molecular simulations (excluding the MD simulations) were conducted using an Apple MacBook Pro equipped with the M4 Pro chip featuring a 14-core CPU, 20-core GPU, and 16-core Neural Engine, along with 24 GB of unified memory and a 512 GB SSD. A high-performance workstation was utilized for the MD simulations. The system was powered by an Intel® Core™ i9-12900KF CPU (3.90 GHz, 16 cores), Intel Corporation, Santa Clara, CA, USA; an NVIDIA GeForce RTX 4090 GPU with 24 GB GDDR6X memory, NVIDIA Corporation, Clara, CA, USA; 64 GB DDR5 RAM, Corsair, Fremont, CA, USA; an 8 TB HDD storage, Western Digital, San Jose, CA, USA; and running the Ubuntu operating system, Canonical Ltd., London, UK.
